# Full-length transcriptome analysis of *Spodoptera frugiperda* larval brain reveals detoxification genes

**DOI:** 10.7717/peerj.12069

**Published:** 2021-08-24

**Authors:** Lei Yang, Binglin Xing, Fen Li, Li Kui Wang, Linlin Yuan, Amosi Leonard Mbuji, Zhengqiang Peng, Farag Malhat, Shaoying Wu

**Affiliations:** 1 Hainan University, Haikou, Hainan, China; 2 Department of Resources Utilization and Plant Protection, College of Resources and Environmental Science, China Agricultural University, Beijing, Beijing, China; 3 The Ministry of Agriculture and Rural Affairs Key Laboratory of Integrated Pest Management of Tropical Crops, Environment and Plant Protection Institute, Chinese Academy of Tropical Agricultural Sciences, Haikou, Hainan, China; 4 Pesticide Residues and Environmental Pollution Department, Agricultural Research Center, Dokki, Giza, Egypt

**Keywords:** *Spodoptera frugiperda*, Resistance, Full-length transcriptome, Detoxification gene

## Abstract

**Background:**

*Spodoptera frugiperda* (J. E. Smith), commonly known as fall armyworm (FAW), is one of the most destructive agricultural pests in the world and has posed a great threat to crops. The improper use of insecticides has led to rapid development of resistance. However, the genetic data available for uncovering the insecticide resistance mechanisms are scarce.

**Methods:**

In this study, we used PacBio single-molecule real-time (SMRT) sequencing aimed at revealing the full-length transcriptome profiling of the FAW larval brain to obtain detoxification genes.

**Results:**

A total of 18,642 high-quality transcripts were obtained with an average length of 2,371 bp, and 11,230 of which were successfully annotated in six public databases. Among these, 5,692 alternative splicing events were identified.

## Introduction

*Spodoptera frugiperda* (J. E. Smith), known as fall armyworm (FAW), belongs to the Noctuidae of Lepidoptera. It is one of the most dangerous invasive pests originated from the tropical and sub-tropical regions of the Americas (Capinera, 2002). FAW possesses a wide range of hosts, including at least 353 plants belonging to 76 families, such as corn, wheat, rice, sorghum and others (Wan et al., 2020), it has caused great damage to crops, thus leading to huge economic losses to agriculture. FAW is an alien invasive pest in China and quickly invaded the Yunnan Province, southern China in December 2018 (Sun et al., 2021; Wu, Jiang & Wu, 2019). At the end of 2020, it had colonized corn fields of 1,338 counties of 27 provinces in China, and the damaged area caused by FAW has reached as high as 11,125–12,780 hectares (Zhou et al., 2021). FAW adult has a strong capability of long-distance migration, and the average flight distance was more than 100 km per night under proper environmental conditions (Tendeng et al., 2019), which aggravating its rapid spreading (Wan et al., 2020). Additionally, the fecundity of FAW is extremely high, the average number of laying eggs per female is 1,052–1,323 (Wan et al., 2020). More exaggeratedly, the maximum number of egg production per female reached 2,000 in Africa (Prasanna et al., 2018). To sum up, the gluttony, strong migration ability and high fertility of FAW have attracted extensive attention. Therefore, it was classified as the top first destructive pest in China (Zhou et al., 2021).

Transcriptome especially the short-reading sequencing technique has long been developed and used as one of the most efficient methods to obtain reference gene sequences, quantify gene expression levels and characterize gene functions not only in vertebrates, but also in invertebrates such as insects (Djebali et al., 2012; Ekblom & Galindo, 2011; Nagalakshmi et al., 2008; Yin et al., 2014, 2016). Although booming advances in technology have been made recently, there were many limitations (Zhou et al., 2010). For instance, this sequencing technology failed to identify genes longer than 5,000 bp upon most occasions, and the excavation of alternative splicing spanning entire transcripts was rather difficult (Koren et al., 2012). Moreover, incorrect annotations were often conducted due to low-quality transcripts. To overcome these limitations, third generation full-length transcriptome based on PacBio single-molecule real-time (SMRT) sequencing technology was developed (Picelli et al., 2013). Compared to the short-reading sequencing technique, SMRT sequencing greatly contributes to our understanding of the physiological processes of objects at a molecular level, such as the availability of accurate gene full-length (maximum > 20,000 bp), identifiable alternative splicing isoforms, and easy to screen novel transcripts.

Insects are naturally under the selection pressure of various pesticides in local ecological context. As a coping strategy, several defensive mechanisms have been formed to enhance the metabolism of chemicals, decrease their toxic effect and ensure their survival during long-term evolution (Despres, David & Gallet, 2007). Among these, metabolic resistance is of vital importance, which involves many detoxification enzymes acting in the biotransformation of hazardous chemicals to non-toxic metabolites. To date, many detoxification gene families have been characterized typically classified into five categories according to their different metabolic phases, among which the ATP-binding cassettes (ABCs) are involved in the transport of a broad variety of substrates across the cell membrane (Atsumi et al., 2012), and carboxylesterases (CESs) hydrolyze an ester *via* the addition of water to form the corresponding alcohol and acid (Wheelock, Shan & Ottea, 2005). In contrast, cytochrome P450s (CYPs) are terminal oxidases involved in drug and steroid hydroxylation reactions (Scott, 1999), and glutathione S-transferases (GSTs) can catalyze the conjugation of glutathione to insecticides or directly metabolize the insecticides to less toxic soluble conjugate or non-catalytically bind to the insecticides (Kostaropoulos et al., 2001), while UDP-glucuronosyl transferases (UGTs) conjugate the uridine diphosphate glucuronic acid to metabolites to decrease hydrophobicity (Li et al., 2018). The roles of these detoxification genes had been well documented in numerous phytophagous pests such as lepidopteran, coleopteran, hemipteran, dipteran, and other species (Enayati, Ranson & Hemingway, 2005; Stevens et al., 2000; Yang et al., 2005). However, the full-length data of detoxification genes are scarce.

Here, we used PacBio SMRT sequencing technology to profile the full-length transcriptome of the FAW larval brain. Next, five main gene families associated with the detoxification function were classified, and the predicted lncRNA targeting these transcripts were further analyzed. These findings undoubtedly provide important information for exploring the scenario of these genes and revealing their functions.

## Materials & Methods

### Insect rearing and sampling

FAW larvae were collected from the corn field in Baoting, Hainan, Southern China (19°14′45.12″N, 109°89′40.40″E) on May 5th, 2019. We established a laboratory colony by feeding the larvae on artificial diet under conditions of 25 ± 1 °C, 14 h:10 h (L:D) photoperiod, and 55 ± 5% relative humidity. The third instar larvae were individually transferred into a small box and reared until pupation. Adults were transferred into a home-made insect raise cage (Patent no. 201921652702.2) after inclusion and fed with 10% hydromel (v/v). Once adequate fourth instar larvae were obtained, we rinsed them in 75% ethanol (v/v) and sterile phosphate-buffered saline (PBS, pH 7.2) twice, respectively. Next, the brain was dissected in PBS containing 1 unit/µL Murine RNase inhibitor (Vazyme, Nanjing, China) under an Olympus SZ61 stereomicroscope (Olympus, Tokyo, Japan). We collected the sample into a 1.5 ml Eppendorf tube for subsequent RNA extraction.

### RNA isolation and SMRT sequencing

We extracted the total RNA of FAW larval brain using TRIzol reagent (Invitrogen, Carlsbad, CA, USA) according to the description of manufacturer and checked the RNA purity (OD 260/280) using Nanodrop 2000 spectrophotometer (NanoDrop Products, Wilmington, DE, USA). Then, RNA degradation and DNA contamination were detected by 1% agarose gel electrophoresis. After a quality inspection, 12 µg RNA was used to gather mRNA molecules by oligo (dT) magnetic beads. We constructed a full length cDNA library using the Clontech SMARTer PCR cDNA synthesis kit (Clontech, Mountain View, CA, USA). The synthesized cDNA was purified by AMPure XP beads (Beckman Coulter, Brea, CA, USA) and sequenced on the PacBio sequel II platform (Pacific Biosciences, Menlo Park, CA, USA) based on the manufacturer’s instructions. Raw data of FAW transcriptome were available from the NCBI SRA database (Project number: PRJNA729608).

### Data processing of full-length transcriptome

We used SMRTlink 6.0 software (Pacific Biosciences, Menlo Park, CA, USA) to process the sequence data. Then, reads containing adaptors, Poly-N, and low-quality from raw data were removed, and the filtered reads were converted into cyclic consensus sequence (CCS) according to the criteria of full passes ≥1 and sequence accuracy >0.9. Full length and non-full length reads were respectively predicted followed by arrow polishing using Quiver. Subsequently, we removed redundant sequences using the CD-hit program and aligned these terms against the FAW reference genome (SRA project number: PRJNA647344) using GMAP (http://research-pub.gene.com/gmap/) (Li & Godzik, 2006). The obtained transcripts were used for the next analysis.

### Transcript annotation and alternative splicing analysis

All transcripts were functionally annotated based on the six databases, NCBI non-redundant protein sequences (NR) database using blastx with e-value <1e^−5^ (Pruitt, Tatusova & Maglott, 2007), Protein family (Pfam) database using Hmmscan, a HMMER-based approach for detecting matches to Pfam families (Finn, Clements & Eddy, 2011; Finn et al., 2009), a manually annotated and reviewed protein sequence (Swiss-Prot) database (Bairoch & Apweiler, 2000), Clusters of Orthologous Groups of proteins (KOG) database (Tatusov et al., 2000), Kyoto Encyclopedia of Genes and Genomes (KEGG) database using Diamond v0.8.36 software with e-value <1e^−5^ (Kanehisa & Goto, 2000), Gene Ontology (GO) database using Blast2GO v2.5 (Conesa et al., 2005; Consortium, 2004), individually. To identify alternative splicing events, transcripts were further processed using Astalavista software with default parameters (Foissac & Sammeth, 2007), and we defined alternative splicing events based on the criteria of previous description (Shen et al., 2021).

### Identification and phylogeny analysis of detoxification genes

Five detoxification gene families, CYP, ABC, CES, GST, and UGT, were screened based on the annotation results and further manually verified. A simple modular architecture research tool (SMART) was used for predicting the conserved domains (Letunic & Bork, 2017). We conducted multiple sequence alignments using Clustal Omega (http://www.ebi.ac.uk/Tools/msa/clustalo/) followed by constructing phylogenetic trees based on the maximum likelihood method using Mega-X software with 1,000 bootstrap values (Kumar et al., 2018), which was further edited and visualized using FigTree v1.4.3 software (Institute of Evolutionary Biology, University of Edinburgh, Edinburgh, UK).

### LncRNA identification, target prediction and motif analysis

We used four analysis tools, Pfam (Finn et al., 2014), Coding-Non-Coding-Index (CNCI) (Sun et al., 2013), Coding Potential Calculator (CPC) (Kong et al., 2007), and Coding Potential Assessment Tool (CPAT) to screen potential lncRNAs (Wang et al., 2013). To ensure the accuracy of these results, transcripts simultaneously recognized by these tools were defined as lncRNAs. In the following, LncTar tool was used to predict the lncRNA-mRNA interactions (Li et al., 2015), and motif analysis was conducted using the online tool MEME (Bailey et al., 2015).

## Results

### Overview of full-length transcriptome data

The 61.50 GB clean data of fall armyworm (FAW) larval brain full-length transcriptome were obtained by SMRT sequencing. A total of 297,682 circular consensus sequences (CCSs) were generated, and 248,554 sequences containing poly-A tail were identified as full length sequences (Table 1). Among these, 234,863 reads (94.49%) was classified as non-chimeric sequences with a mean read length of 2371 bp. Next, redundant sequences within different transcript clusters were removed using CD-hit program, and 18,642 high quality non-redundant transcripts were identified and used for subsequent analysis.

**Table 1:** Overview of FAW larval brain full-length transcriptome.

Category	Number
Circular consensus sequences (CCS)	297,682
Full-length sequences	248,554
Non-chimeric full-length sequences with Poly-A Tail	234,863
Average read length	2,371
High quality non-redundant transcripts	18,642
Alternative splicing events	5,692
Predicted lncRNA	1,319
Transcripts with functional annotation	11,230
Transcripts with ORF	11,013

### Transcript annotation

**Figure 1:**
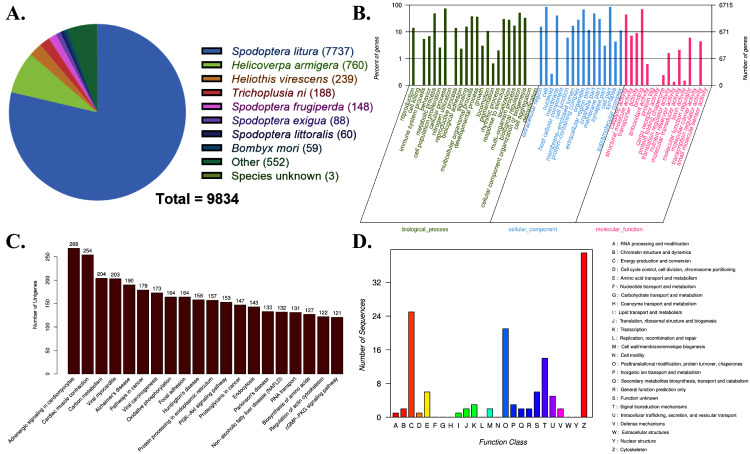
Full-length transcript annotation of FAW larval brain. (A) Homologous species distribution of transcripts in NR database. (B) GO functional categorization of transcripts. (C) Top 20 KEGG functional classifications of transcripts. (D) COG functional classification of transcripts.

To reveal the function of 18,642 transcripts, we annotated these terms on six databases. Notably, 9,834, 7,805, 6,821, 6,715, 5,513 and 219 transcripts were annotated in NR, Pfam, Swiss-Prot, GO, KEGG, and KOG databases, respectively. In total, we obtained 11,230 transcripts with valid functional prediction in at least one database. What stood out in Fig. 1A was that 7,737 homologous transcripts (68.90%) best matched the *S. litura*, and the top seven hits belonged to the Noctuidae of Lepidoptera based on the annotation results in NR database, showing high similarity. Next, all transcripts were annotated in GO database and 6,715 terms were classified into three GO categories, biological process, cellular component, and molecular function (Fig. 1B). In biological processes, we found that most of the transcripts were associated with cellular processes followed by metabolic processes and biological regulation. In contrast, the transcripts involved in cell and cell parts were the most abundant terms in the cellular component category. Additionally, in molecular function, the top three annotated transcripts having binding, catalytic and transporter activity were respectively identified (Fig. 1B). We also sorted these transcripts into different groups according to their participation in KEGG metabolic pathways. Among the top 20 annotated terms, the most enriched categories pertained to adrenergic signaling in the cardiomyocyte pathway (268) followed by the cardiac muscle contraction pathway (254) (Fig. 1C). Finally, we annotated 219 transcripts in KOG databases, transcripts participating in “Cytoskeleton”, “Energy production and conversion” and “Post-translational modification, protein turnover, chaperones” were the top three abundant terms (Fig. 1D).

### CYPs

**Figure 2:**
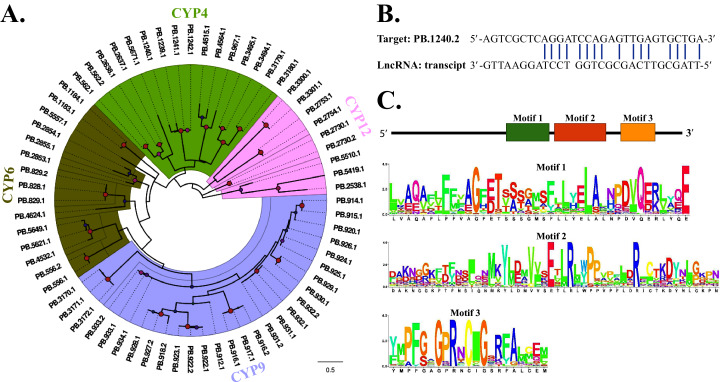
Phylogenetic construction, lncRNA target prediction and motif analysis of CYPs. (A) Phylogenetic tree of 84 FAW CYP transcripts. The evolutionary tree was constructed based on the maximum likelihood method using the program Mega X with 1,000 bootstrap values. Forty-eight different amino acid substitution models were tested and “LG+G” was the best model. (B) The binding sites of lncRNA targeting transcript “PB.1240.2”. LncTar was used to predict RNA targets of lncRNAs with a cutoff of −0.1 normalized deltaG. (C) Motif analysis of 84 CYPs. We used MEME tool to identify the conserved motifs.

Based on the functional annotation, 84 transcripts were characterized as CYPs with a mean length of 2,146 bp, further being manually confirmed in NR database. Among these, 64 transcripts with full length were identified. A phylogenetic tree based on the conserved “CYP domain” was constructed, and results showed that these transcripts were distributed in four clades, CYP4, CYP6, CYP9 and CYP12. Among these, CYP9 concluding 28 transcripts made up the largest clade (Fig. 2A), indicating their functional redundancy. We further identified a lncRNA targeting the transcript “PB.1240.2”, and the predicted binding site was located in the CDS region (Fig. 2B). Besides, the conserved motif analysis presented an overview of the conserved amino acid residues among 84 transcripts (Fig. 2C).

### ABCs

**Figure 3:**
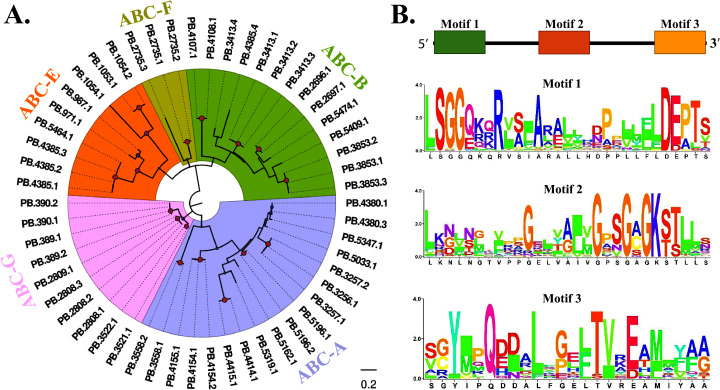
Phylogenetic and motif analysis of ABCs. (A) Phylogenetic tree of 54 FAW ABC transcripts. The evolutionary tree was constructed based on the maximum likelihood method using the program Mega X with 1,000 bootstrap values. Forty-eight different amino acid substitution models were tested and “LG+G” was the best model. (B) Motif analysis of 54 ABCs. We used MEME tool to identify the conserved motifs.

Fifty-four transcripts encoding ABCs were obtained from the transcriptome of the FAW larval brain, and nine of which did not contain full length. The mean length of 49 ABCs was 4,074 bp. In typical cases, ABCs were embedded with one or more “ABC transmembrane domain”, we thus constructed a phylogenetic tree based on the conserved domains. From the data in Fig. 3A, it was apparent that 54 transcripts clustered into five clades, namely ABC-A, ABC-B, ABC-E, ABC-F, and ABC-G. Among these, ABC-A occupied the majority, which contained 18 members. None of the lncRNAs were predicted to bind to the 54 ABC members. Subsequently, several conserved amino acid sites were identified based on the motif analysis (Fig. 3B).

### CESs

**Figure 4:**
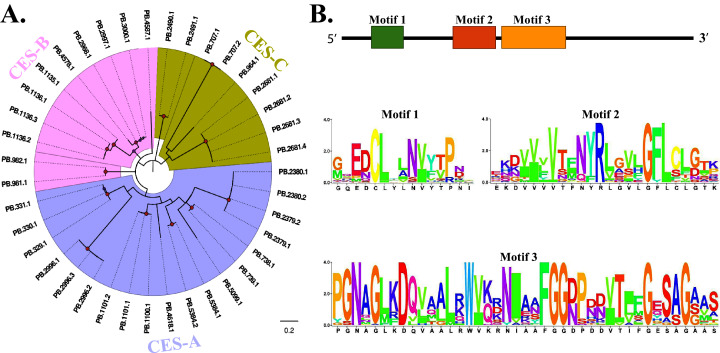
Phylogenetic and motif analysis of 46 CESs. (A) Phylogenetic tree of 46 FAW CES transcripts. The evolutionary tree was constructed based on the maximum likelihood method using the program Mega X with 1,000 bootstrap values. Forty-eight different amino acid substitution models were tested and “LG+G+I” was the best model. (B) Motif analysis of 46 CESs. We used MEME tool to identify the conserved motifs.

After removal of redundant sequences, 46 transcripts were annotated as CESs and half of these were equipped with full length with a mean length of 2,493 bp. To classify these transcripts into different categories, a phylogenetic analysis based on the “CES domain” was constructed. What stood out in Fig. 4A was that 46 CESs clustered into three branches, namely CES-A, CES-B and CES-C. Again, no predicted lncRNAs targeting these transcripts were disclosed. According to the results of motif analysis, we identified several amino acid residues highly conserved among the CES transcripts (Fig. 4B).

### GSTs

**Figure 5:**
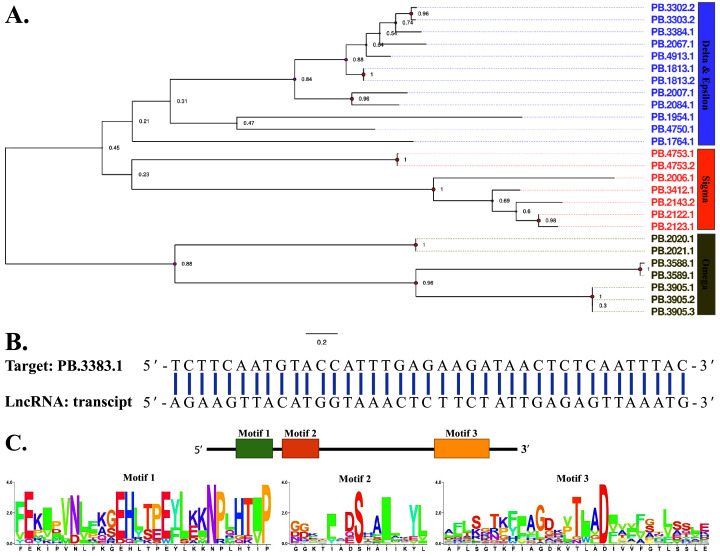
Phylogenetic construction, lncRNA target prediction and motif analysis of GSTs. (A) Phylogenetic tree of 33 GSTs. The evolutionary tree was constructed based on maximum likelihood method using the program Mega X with 1,000 bootstrap values. Forty-eight different amino acid substitution models were tested and “LG+G” was the best model. (B) The binding sites of lncRNA targeting transcript “PB.3383.1”. LncTar was used to predict RNA targets of lncRNAs with a cutoff of −0.1 normalized deltaG. (C) Motif analysis of 33 GSTs. We used MEME tool to identify the conserved motifs.

According to the functional annotation of transcriptome data in six databases, 33 transcripts were identified as GSTs, in which 18 terms were available of full length and the mean length was 1,781 bp. GST was embedded with at least one “GST domain” in the N-terminal and one “GST domain” in the C-terminal, respectively, we thus assigned these transcripts into different groups based on the evolutionary relationship of GST domains. From the graph below, we could see that three clusters, including “Delta and Epsilon”, “Sigma” and “Omega” gathered together, indicating their functional diversity (Fig. 5A). The lncRNA target prediction revealed a regulatory lncRNA could bind to the CDS of “PB.3383.1” transcript (Fig. 5B). Additionally, the results obtained from motif analysis of 33 transcripts were shown in Fig. 5C, and many conserved sites were also revealed.

### UGTs

**Figure 6:**
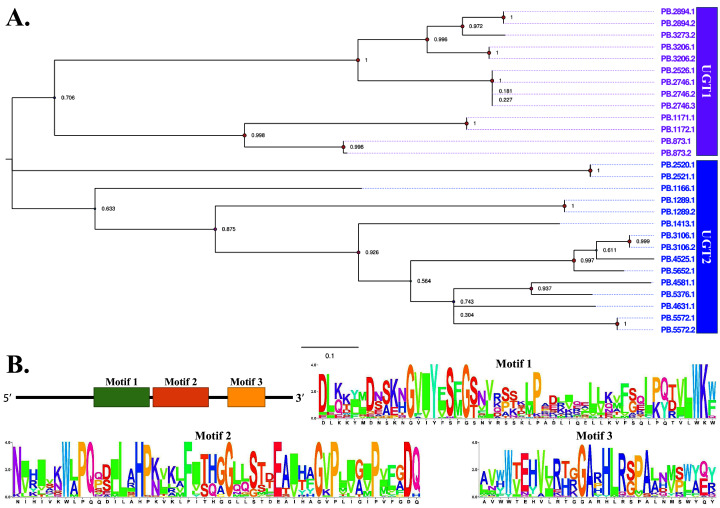
Phylogenetic and motif analysis of UGTs. (A) Phylogenetic tree of 31 UGTs. The evolutionary tree was constructed based on maximum likelihood method using the program Mega X with 1,000 bootstrap values. Forty-eight different amino acid substitution models were tested and “LG+G+I” was the best model. (B) Motif analysis of 31 UGTs. We used MEME tool to identify the conserved motifs.

Thirty-one transcripts containing “UGT domain” were annotated as UGTs and among these 19 full-length sequences were obtained. The mean length of the fully sequenced transcripts was 2,104 bp. Phylogenetic analysis revealed that these transcripts evolved into two branches, “UGT1” and “UGT2” (Fig. 6A). Besides, no lncRNAs were identified as the regulator of UGTs. As shown in Fig. 6B, there were several conserved amino acid residues identifiable from 31 UGTs.

## Discussion

The full transcriptome analysis of FAW larval brain revealed that 18,642 high-quality transcripts, 11,230 of which were categorized into different functional groups based on homologous blast in six public databases. From the results, we could see that most of the transcripts showed a higher homology with those of *S. litura*, a closely related species of FAW, which can provide a valuable reference for subsequent gene function characterization of FAW. Moreover, GO enrichment analysis, KEGG metabolic pathway, and KOG annotations also provided us a reference for the function of these transcripts. Alternative splicing events form diverse gene variants, and 5,692 alternative splicing events were identified in this assay, possibly accounting for the diversification of gene functions of these transcripts.

The next section of the survey was concerned with detoxification genes. An abundance of evidence has accumulated over the last few decades, indicating that insect herbivores have evolved a set of detoxification genes to detoxify chemicals (Rane et al., 2019). In typical cases, the detoxification enzymes include CYPs, ABCs, CESs, GSTs, UGTs and so on (Georghiou, 2012). As one of the largest superfamily, CYPs commonly exist in insects while the number varies a lot. For instance, there was a total of 143 genes in *Tribolium castaneum* (Zhu et al., 2013). Comparatively, only 38 CYPs were identified in *Pediculus humanus humanus* (Lee et al., 2010). A previous study has examined CYPs of FAW by whole genome-wide sequencing and finally identified 152 CYP gene clusters (Gui et al., 2020). These results also corroborated the ideas of Xiao et al. (2020), who discovered 169 FAW CYPs with widespread expression at different developmental stages (Xiao et al., 2020). In this study, only 84 transcripts encoding CYPs were annotated in full-length transcriptome data of the FAW larval brain, showing that numerous CYPs were not specifically expressed in the brain. Phylogenetic analysis clustered these transcripts into four clans, among these 15 unique terms were classified into the CYP6 clade. It has been widely known that CYP6 family members act in detoxifying pesticides (Ibrahim et al., 2016; Shi et al., 2018; Yang et al., 2020). Therefore, our findings will provide an important reference for exploring the molecular mechanism of FAW CYPs in detoxification. The motif analysis displayed several conserved amino acid sites among 84 CYPs possibly crucial for its function.

Relative to CYPs, ABCs are a set of newly identified detoxification enzymes, among which the ABC-C2 genes are largely responsible for transport of activated Cry toxins into the cell, and the insecticide resistance is derived from loss of function in ABC-C2 (Merzendorfer, 2014). Recently, the whole genome sequencing of various insect species provides a great convenience to identify the ABC gene family. For example, 104, 73, 56 and 53 ABCs were characterized in *Tetranychus urticae* (Dermauw et al., 2013), *T. castaneum* (Broehan et al., 2013), *Drosophila melanogaster* (Liu et al., 2011), and *Bombyx mori* (Liu et al., 2011), respectively. A recent study revealed that 57 ABCs were expressed in FAW combining genome and transcriptome analyses (Gui et al., 2020). In this assay, 54 transcripts were identified, which was in accordance with the previous results. These ABCs were categorized into five functional groups, ABC-A, ABC-B, ABC-E, ABC-F and ABC-G. Several evidences have shown that ABCs are closely related to insecticide resistance in insects. For instance, in *Trichoplusia ni*, a significant increase in transcript levels in ABC-B genes was recorded after exposure to deltamethrin (Simmons et al., 2013). Besides, five ABC-B and 13 ABC-C genes of *Anopheles gambiae* were thought to be responsible for detoxification (Roth et al., 2003). Many conserved amino acid residues were also identified among 54 ABCs by motif analysis, implying the potential targets for developing novel pesticides.

As a member of the large carboxylesterase family, CESs are also implicated in the detoxification of various xenobiotic compounds, especially the organophosphate insecticides (Wheelock, Shan & Ottea, 2005). CESs have been well characterized in many flies and aphids, such as *Lucilia cuprina* (Jackson et al., 2013), *D. melanogaster* (Cui et al., 2015), *Sitobion avenae* (Xu et al., 2014), *Aphis gossypii* (Cao et al., 2008), *Myzus persicae* (Devonshire, Moores & Ffrench-Constant, 1986). Many studies supported that CESs act in alleviating insecticidal efficacy through gene amplification or up-regulated their transcriptional level (Hemingway, 2000). Here, we annotated 46 FAW CESs embedded with many conserved amino acid residues, more than the 30 terms previously reported (Gui et al., 2020), and then assigned these terms into three clades from an evolutionary perspective. We thus speculated that these CESs may account for the rapid development of FAW resistance to pesticides.

GSTs can produce resistance to all main classes of insecticides. Advances in genomic and transcriptomic sequencing technologies triggered an exhaustive identification of GSTs in many insects, including 11 crop pests and four species of mosquito (Pavlidi, Vontas & Van Leeuwen, 2018). Our present investigation identified 33 GSTs distributed in three clades equipped with many conserved amino acid residues. In contrast, a recent study reported 29 GSTs of FAW combining genomic and transcriptomic analyses (Gui et al., 2020). Among these, 15 genes were remarkably down-regulated after pesticide treatment. Also, in *S. litura*, a closely related species of FAW, 37 transcripts were annotated and their expression levels after chlorpyrifos treatment were analyzed (Zhang et al., 2016). These findings undoubtedly provided important references for further functional study of GSTs in detoxification.

UGTs also belong to the relatively newly identified classes. As a kind of detoxification enzyme, UGTs widely exist in lepidopteran insects, including *B*. *mori* (Huang et al., 2008), *S. litura* and many other species (Shen et al., 2021; Shi et al., 2019). In typical cases, UGTs are classified into two distinct subfamilies, UGT1 and UGT2. Compared with the 49 UGTs previously detected in FAW genome and transcriptome (Gui et al., 2020), a small number of UGTs representing 31 transcripts grouped into two families were annotated in this study. It was noted that 17 FAW UGTs showed significant differential expression after pesticide treatment (Gui et al., 2020). Similarly, 10 UGTs were remarkably up-regulated in both indoor and field indoxacarb-resistant strains of *S. litura* (Shi et al., 2019). Taken all these findings together, we speculated that the 31 UGT transcripts aid in the detoxification process. We also identified several conserved amino acid residues in these UGTs, providing promising targets for developing novel pesticides.

LncRNAs are implicated in various biological functions such as protein synthesis, RNA transport and *etc*., one of the most important roles in which is the gene expression regulation through gene silencing (Wang & Chang, 2011). The full transcriptome sequencing provides an alternative method to predict lncRNAs with the potential binding ability to transcripts. Thus, we analyzed the lncRNAs targeting the transcripts of five main detoxification gene families, and the results, while preliminary, suggested that only two lncRNAs potentially bond to the transcript PB.1240.2, a member of CYPs, and the transcript PB.3383.1, a member of GSTs, respectively. These findings may help us understand the underlying molecular mechanism that lncRNAs regulate detoxification genes.

## Conclusions

Full-length transcriptome of the FAW larval brain was obtained based on the PacBio SMRT sequencing and analyses involving gene annotation, lncRNA prediction and alternative splicing events were conducted. A further in-depth study examined the five detoxification gene families, which provided an abundant genetic resource and laid a solid foundation for revealing the resistance mechanisms of FAW to pesticides.

## Supplemental Information

10.7717/peerj.12069/supp-1 Supplemental Information 1 Annotation of identified five detoxification gene families.

## Additional Information and Declarations

### DNA Deposition

The following information was supplied regarding the deposition of DNA sequences: The raw data and assembled transcriptome of full-length transcriptome of *Spodoptera frugiperda* larval brain described here are available at NCBI SRA: PRJNA729608.

### Data Availability

The following information was supplied regarding data availability: The raw data are available in the Supplemental Table.
